# Clusterin silencing restores myoblasts viability and down modulates the inflammatory process in osteoporotic disease

**DOI:** 10.1186/s12967-019-1868-5

**Published:** 2019-04-10

**Authors:** S. Pucci, C. Greggi, C. Polidoro, M. C. Piro, M. Celi, M. Feola, E. Gasbarra, R. Iundusi, F. Mastrangeli, G. Novelli, A. Orlandi, U. Tarantino

**Affiliations:** 1 Department of Biomedicine and Prevention, Tor Vergata University of Rome, Via Montpellier 1, 00133 Rome, Italy; 2 Department of Clinical Sciences and Translational Medicine, University of Rome Tor Vergata, Via Montpellier 1, 00133 Rome, Italy; 3 Department of Orthopedics and Traumatology, “Policlinico Tor Vergata” Foundation, Viale Oxford 81, 00133 Rome, Italy; 4 Department of Experimental Medicine and Surgery, University of Rome “Tor Vergata”, Via Montpellier 1, 00133 Rome, Italy

**Keywords:** Clusterin (CLU), Osteoporosis, Osteoarthritis, Sarcopenia, Muscle waist, Osteoporosis marker

## Abstract

**Background:**

Targeting new molecular pathways leading to Osteoporosis (OP) and Osteoarthritis (OA) is a hot topic for drug discovery. Clusterin (CLU) is a glycoprotein involved in inflammation, proliferation, cell death, neoplastic disease, Alzheimer disease and aging. The present study focuses on the expression and the role of CLU in influencing the decrease of muscle mass and fiber senescence in OP-OA condition.

**Methods:**

*Vastus lateralis* muscle biopsies were collected from 20 women with OP undergoing surgery for fragility hip fracture and 20 women undergoing arthroplasty for hip osteoarthritis.

**Results:**

We found an overexpression of CLU in degenerated fibers in OP closely correlated with interleukin 6 (IL6) and histone H4 acetylation level. Conversely, in OA muscle tissues we observed a weak expression of CLU but no nuclear histone H4 acetylation. Ex vivo studies on isolated human myoblasts confirmed CLU overexpression in OP as compared to OA (p < 0.001). CLU treatment of isolated OP and OA myoblasts showed: modulation of proliferation, morphological changes, increase of histone H4 acetylation and induction of myogenin (MYOG) activation in OP myoblast only. In OP condition, functional knockdown of CLU by siRNA restores proliferative myoblasts capability and tissue damage repair, carried out by an evident upregulation of Transglutaminase 2 (TGM2). We also observed downmodulation of CX3CR1 expression with consequent impairing of the inflammatory infiltrate recruitment.

**Conclusions:**

Results obtained suggest a potential role of CLU in OP by influencing myoblasts terminal differentiation, epigenetic regulation of muscle cell differentiation and senescence. Moreover, CLU silencing points out its role in the modulation of tissue damage repair and inflammation, proposing it as a new diagnostic marker for muscle degeneration and a potential target for specific therapeutic intervention in OP related sarcopenia.

## Background

Osteoporosis (OP), Osteoarthritis (OA) and sarcopenia are multifactorial diseases with a clear genetic component, in which both immune response and chronic inflammation play an important role. The pathogenic process of these degenerative diseases leads, eventually, to the degeneration of cartilage, subchondral bone-to-bone abnormalities and severe impairment of joint function and, in the case of OP, also to sarcopenia. In particular, sarcopenia is an aging-induced generalized pathological condition characterized by loss of muscle mass and age-related functions [1, 2]. The conditions leading to muscle loss involve different intracellular signaling pathways, apoptosis, mitochondrial dysfunctions [3] and the alteration of lipidic pathways. The development and homeostasis of different systems such as the nervous, muscular and bone relies on the presence of key regulatory molecules like growth factors, soluble mediators and their respective receptors. A crucial role in aging is played by proinflammatory factors such as interleukin 6 (IL6) that mediates cell–cell interactions. Aging and the subsequent atrophy of muscle fibers lead to a reduction of mechanical bone stimuli exercised through the tendons, which regulate bone development and remodelling, thus bringing to a degenerative process such as OA and OP [4]. In this context, Clusterin (CLU) is a new pleiotropic factor potentially involved in the stimulation of inflammatory cytokines such as IL6 and lipid metabolism, cell differentiation, tissue remodeling and neoplastic diseases [5–7].

Emerging evidences suggest that CLU plays an important role in muscle and bone homeostasis. CLU is an heterodimeric disulfide-linked protein of ~ 75–80 kDa involved in several physiological processes including lipid transportation in serum, cellular senescence, aging and various age-related diseases, like neurodegeneration, inflammation, vascular damage, diabetes and tumourigenesis [8–17]. Two different CLU transcripts, derived from alternative splicing, have been identified: one coding for a nuclear form (nCLU, 50–55 kDa) and a second one coding for a highly glicosilated cytoplasmatic form (sCLU, 40 kDa) found also in biological fluid. In particular sCLU is an intra- and extracellular chaperon stress inducible protein and it has also been functionally implicated in signalling pathways that regulate development, differentiation and apoptotic cell death. CLU polymorphisms were recently found to be associated with Alzheimer’s disease [18–20]. Existing studies referring to sCLU in degenerative joint diseases are limited and its role in these pathologies is still obscure. The main purpose of the present study was therefore to characterize new pathways and molecular targets involved in the onset and progression of OP and OA. We focused on the role of CLU, which is involved also in calcium metabolism [21], to clarify its function in these pathologies and its potential action in reduction of muscular mass leading to sarcopenia and its possible involvement in the inflammatory process that characterizes OP and OA condition [22]. Through the in vitro experiments, we analyzed the effect of CLU on proliferation, on DNA acetylation levels and on myogenin (MYOG) activation, the main marker of myoblasts differentiation. Furthermore, we characterized the effect of CLU silencing by siRNA on: proliferation, expression of genes and proteins involved in tissue damage repair and in the initiation of the inflammatory process.

## Methods

### Patients

We enrolled 40 patients who underwent hip surgery in the Orthopedic Department of “Tor Vergata” University Hospital, between 2016 and 2018. Specifically, we enrolled 20 osteoporotic older women who underwent hip arthroplasty for subcapital fractures of the femur (80.58 ± 6.3 years; mean ± standard error of the mean (SEM)) and 20 osteoarthritic older women (67.63 ± 8 years) who underwent hip arthroplasty for OA. Exclusion criteria were history of cancer, myopathies or other neuromuscular diseases or chronic administration of corticosteroid for autoimmune diseases (more than 1 month), diabetes, alcohol abuse, and HBV, HCV, or HIV infections. The main characteristics of OP and OA patients are described in Table 1. All experiments described in the present study were approved by the ethics committee of “Policlinico Tor Vergata” (approval reference number # 85/12). All experimental procedures were carried out according to The Code of Ethics of the World Medical Association (Declaration of Helsinki). Informed consent was obtained from all patients prior to surgery. Specimens were handled and carried out in accordance with the approved guidelines.

**Table 1:** Main characteristics of OP and OA patients

	OP	OA
Age	80.58 ± 6.3 years	67.63 ± 8 years
BMI	24.56 ± 4.6	27.32 ± 4.4
Menopause age	42.89 ± 1.3	43.68 ± 1.4
T-score (L1–L4)	− 2.8 ± 0.7	− 0.75 ± 0.5
T-score (femoral neck)	− 3 ± 0.6	− 0.67 ± 0.11
HHS	–	47.26 ± 5
K–L score	0–1	3/4

### Bone mineral density evaluation (DXA)

DXA was performed with a Lunar DXA apparatus (GE Healthcare, Madison, WI, USA). Lumbar spine (L1–L4) and femoral (neck and total) scans were performed, and bone mineral density (BMD) was measured according to manufacture’s recommendations [23]. Dual-energy X-ray absorptiometry measures BMD (in grams per square centimeter), with a coefficient of variation of 0.7%. For patients with fragility fractures, BMD was measured on the uninjured limb. For OA patients, measurements were performed on the non-dominant side, with the participants supine on an examination table with their limbs slightly abducted [24]. DXA exam was performed 1 day before surgery for OA patients, and 1 month after surgery for OP patients. The results were expressed as T-scores.

### Harris hip score (HHS)

The Harris hip score (HHS) was measured to evaluate the level of joint dysfunctionality in OA patients. It includes 4 sections. Pain—scoring between 0 and 44 points. Function—up to 47 points divided into walking functions (up to 33 points), and daily activities (up to 14 points). Absence of deformity 4 points, and movement range 5 points [25].

### Radiological evaluation

Hip x-rays were performed in order to check the fracture or to assessed hip OA. Kellgren-Lawrence scale was used in order to determine the severity of OA. The Kellgren and Lawrence system is a method of classifying the severity of OA using five grades. This classification was proposed by Kellgren et al. [26] in 1957. It includes: grade 0 if no radiographic features of OA are present; grade 1 if doubtful joint space narrowing (JSN) and possible osteophytic lipping; grade 2 if definite osteophytes and possible JSN on anteroposterior weight-bearing radiograph; grade 3 if multiple osteophytes, definite JSN, sclerosis, possible bony deformity; grade 4 if large osteophytes, marked JSN, severe sclerosis and definite bony deformity. Two orthopaedists independently assessed all radiographs. Patients with a grade of K−L ≥ 2 were considered osteoarthritic.

### Sampling

During open surgery for hip arthroplasty, muscle biopsies were taken from the upper portion of the *vastus lateralis*. Sample withdrawals were performed for histological analysis excluding macroscopic alteration of skeletal muscle biopsy as necrosis areas.

### Histology

Muscle biopsies were fixed in 4% paraformaldehyde for 24 h and paraffin embedded. 3 μm sections were stained with hematoxylin and eosin (H&E) and subsequently the histomorphometric evaluations were carried out independently by two pathologists. Specifically, both the diameter of 200 muscle fibers and the number of fibers per μm^2^ were measured for each sample by using digital image (iScan Coreo—ImageView software, Ventana, Roche, USA). In order to assess fibers atrophy, a minimum of 200 muscle fibers per biopsy have been evaluated, comparing minimum transverse diameter and cross-sectional area of type I and type II fibers for relative prevalence. A threshold diameter lower than 30 μm characterized atrophic fibers.

### Immunohistochemistry (IHC)

Serial 5 μm thick sections from formalin-fixed and paraffin-embedded specimens were immunostained for IL6, Clusterin β, Acetyl histone H4 and Myosin-slow (Table 2). After washings, reactions were revealed by HRP-conjugated streoptoavidin method and diaminobenzidine (DAB) incubation. Tissue staining was semi-quantitatively graded for intensity from negative (0) to strong (+++). Slides were independently examined by two pathologists, unaware of the clinical data and molecular results. Moreover, all cases were digitally scanned by iScan (BioImagene, Now Roche-Ventana) with Scanning Resolution 0.46 μm/pixel at ×20. The IHC signals were measured using an automated image analyzing system (MECES). Scoring of immunoreactivity was statistically analyzed by Mann–Whitney’s U test. Twenty sections were stained and analyzed for each experimental group.

**Table 2:** Primary antibodies used for immunohistochemistry (IHC) analysis

Antibody	Characteristics	Dilution
IHC		
Anti-IL6	Mouse monoclonal, R&D Systems	1:20
Anti-Cluβ	Goat polyclonal, Santa Cruz Biotechnology	1:100
Anti-acetyl histone H4	Rabbit polyclonal, Upstate	1:75
Anti-myosin slow	Mouse monoclonal, Sigma-Aldrich	1:100

### Cell culture and CLU conditioning

Human myoblast cells were extracted ex vivo and grown on gelatin matrix (Fluka) in complete growth medium supplied with 15% FBS, insulin 1 mg/ml, FGF 5 μg/ml and EGF 10 μg/ml. For CLU treatment, myoblasts were seeded in 96 well multi-wells at 8000 cell/cm^2^, in 6 well multi-wells at 9000 cell/cm^2^ and in 4 well Lab. Tek II Chamber slides at 8000 cell/cm^2^, in complete growth medium (F14 + 15% FBS). After an overnight culture, medium was removed and human recombinant CLU (Endogen) was added at a final concentration of 2 μg/ml for 6 days. Untreated cells were used as control. After 3 and 6 days from treatment cells were counted through trypan blue method. After 48 h, cells were collected for RNA extraction and after 72 h cells were fixed in formalin 10% for ICC analysis.

### SiRNA transfection

In this study, OP and OA myoblasts were silenced for CLU gene. Double-strand purified RNAs pre-designed from Sigma-Aldrich (Milan, Italy) with specific sequences for CLU gene were used: sense 5′-GGAUGAAGGACCAGUGUGAdTdT-3′ and antisense 5′-UCACACUGGUCCUUCAUCCdTdT-3′. Non-specific sequences were used as transfection control (scramble): sense 5′-UUCUCCGAACGUGUCACGUdTdT-3′ and antisense 5′- ACGUGACACGUUCGGAGAAdTdT-3. Cells were seeded in 96 well multi-wells at 8000 cell/cm^2^, in 6 well multi-wells at 9000 cell/cm^2^ and in 4 well Lab. Tek II Chamber slides at 8000 cell/cm^2^, in complete growth medium (F14 + 15% FBS); after an overnight culture, when the cell confluence was at 60–70%, it proceeded with transfection through Lipofectamine^®^ 2000 Transfection Reagent (Invitrogen, Thermo Fisher Scientific), following the guidelines indicated by the company. Lipofectamine and siRNAs (33 nM final concentration) were diluted in Opti-MEM (Gibco-BRL) and incubated for 20 min at room temperature before being added to cells. At the end of the incubation (6 h), transfection complex was removed and complete growth medium was added to cells. After 24, 48 and 72 h cells were counted through trypan blue method. After 48 h from transfection, cells were collected for RNA extraction and after 72 h cells were fixed in formalin 10% for ICC analysis. The efficiency of silencing was confirmed through RT-PCR (Fig. 5).

### RNA extraction, RT-PCR and qRT-PCR

Total RNA was isolated from treated and untreated cells using Tri Reagent (Ambion), according to the manufacturer’s instructions. RNA quantification was performed using spectrophotometry. Reverse transcription of total RNA (1 μg for each myoblast group) was performed with Gene Amp RNA PCR Kit (Applied Biosystems) using Random Examers as primers to cDNA synthesis. β2-microglobulin housekeeping gene was amplified as control. Ethidium-bromide stained 2% agarose gel was run at 100 V and acquired by scanning system. For real time PCR (qRT-PCR) RNA was treated with DNase (2U/ml) and back transcribed with “High Capacity cDNA reverse transcription”. In this study Sybr Green was utilized (Applied Biosystems). GAPDH was used as standard. Each analysis was performed in triplicate. Primers used for cDNA amplification are listed in Table 3.

**Table 3:** Sequences of primers used for RT-PCR and qRT-PCR

Gene	Primer sequences	T annealing (°C)
CLU (RT-PCR)	Sense: 5′-GTGCAATGAGACCATGATGG-3′ Antisense: 5′-CAGGTAGTGGTAGGTATCCT-3′	55
sCLU (qRT-PCR)	Sense: 5′-ATTCTCATCGCTTTGGAAGG-3′ Antisense: 5′-AGACATCAGGGGAGACTTTA-3′	58
TGM2 (RT-PCR)	Sense: 5′-GAGGAGCTGGTCTTAGAGAGG-3 ´ Antisense: 5′-CGGTCACGACACTGAAGGTG-3 ´	62
β2-microglobulin (RT-PCR)	Sense: 5′-CTGGAACGGTGAAGGTGACA-3′ Antisense: 5′-AAGGGACTTCCTGTAACAATGCA-3′	60
GAPDH (qRT-PCR)	Sense: 5′-ACGGATTTGGTCGTATTGG-3′ Antisense: 5′-GATTTTGGAGGGATCTCGC-3′	60

### Immunocytochemistry (ICC)

Human myoblast cells extracted ex vivo were fixed in formalin 10% to perform ICC analysis, as reported above. Primary antibodies used are listed in Table 4. Secondary antibody and following reagents (HRP-conjugated streoptoavidin) were added. After washing, slides were incubated with diaminobenzidine (DAB) and counterstained with haematoxylin.

**Table 4:** Primary antibodies used for immunocytochemistry (IHC) analysis

Antibody	Characteristics	Dilution
ICC		
Anti-Cluβ	Goat polyclonal, Santa Cruz Biotechnology	1:100
Anti-NFKB	Mouse monoclonal, Santa Cruz Biotechnology	1:100
Anti-acetyl histone H4	Rabbit polyclonal, Upstate	1:300
Anti-CX3CR1	Rabbit polyclonal, Mo Bi Tec Molecular Biologische Technologie	1:150
Anti-MYOG	Rabbit monoclonal, AbCam	1:200

### Statistical analysis

All values provided in the text and figures are means of three independent experiments ± standard deviations (SD). The positive stain in IHC and ICC was evaluated by two independent observers. Unpaired t-tests were performed to assess inter-group statistical differences. Differences were considered statistically significant for p < 0.05.

## Results

### Clinical evaluation

The OP group included 20 patients with fragility hip fracture, T-score ≤ − 2.5 SD and K–L score from 0 to 1. The OA group included 20 patients with radiographic evidence of hip OA with a K–L score 3 or 4 and T-score ≥ − 2.5 SD. There was no discrepancy for age, sex and comorbidities in the two groups. Specifically, no patient showed oncological, genetic or neurological diseases, whereas more of 80% of patients were affected by hypertension. Body Mass Index (BMI) mean value of OA patients was significantly higher than BMI mean value of OP group (mean value 27.32 ± 4.4 vs 24.56 ± 4.6, p < 0.001) (Table 1). These data confirmed the frequently overweight condition of OA patients [27].

### IL6 expression and localization in skeletal muscle tissue

**Fig. 1:**
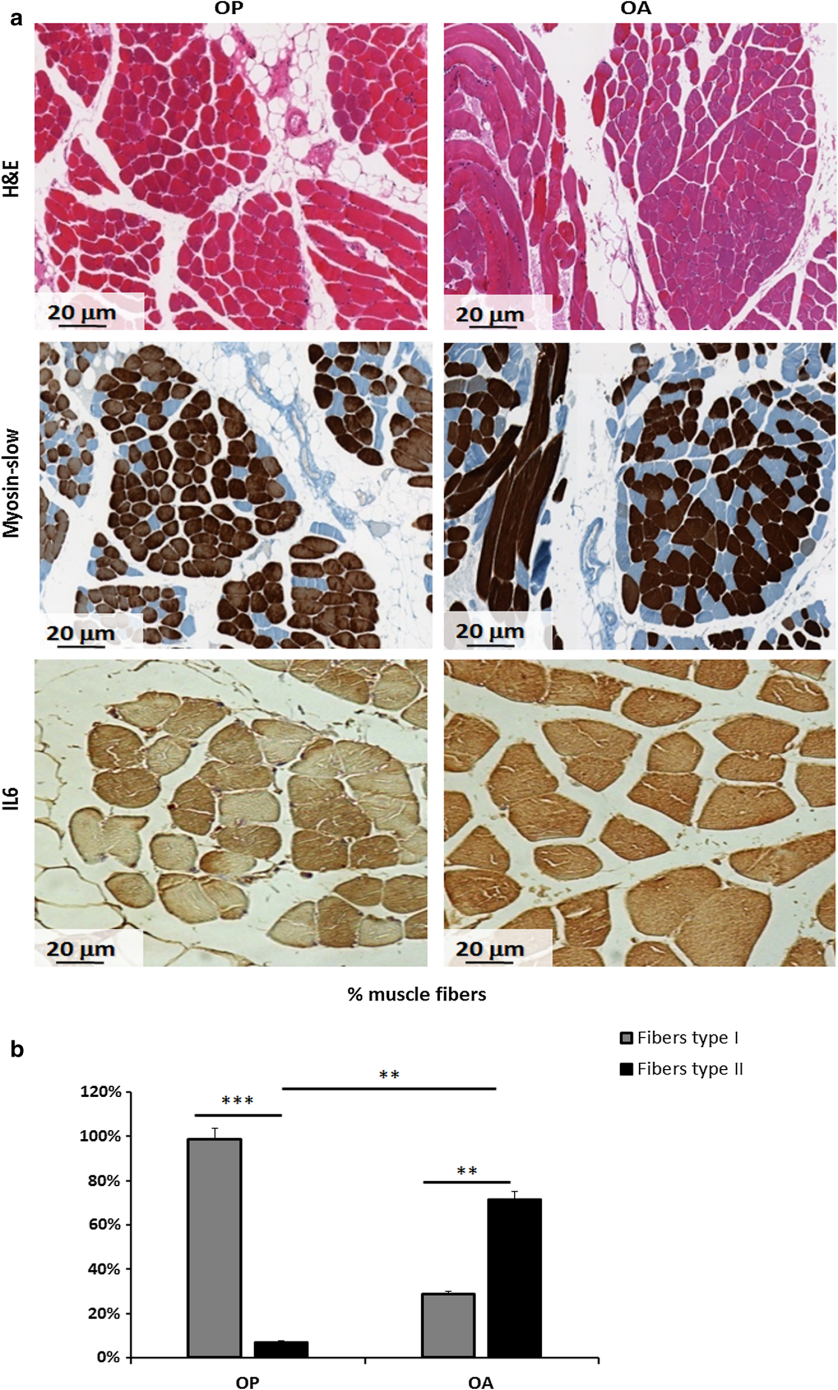
Histomorphometry and IL6 expression analysis in muscle tissues of OP and OA patients: **a** Ematoxilin–eosin (H&E), myosin slow and IL6 staining of muscle tissues from OP and OA patients; **b** percentage of type I and II muscle fibers in OP and OA tissues. This observation supports the best quality of OA muscle as compared to OP (**p < 0.01; ***p < 0.001)

Morphometric analysis on all tissues from OP and OA patients were performed through H&E staining and using the antibody anti-myosin slow characterizing type 1 muscle fibers (Fig. 1a). As shown, it was found that muscle quality appeared to be better in OA patients, where the percentage of fast-twitch type II fibers is higher than OP patients, that show a higher percentage of slow-twitch type I fibers (p < 0.01) (Fig. 1b).

In order to correlate the inflammatory microenvironment to CLU, we observed IL6 expression in the same tissues by IHC. In muscles tissues from OP patients IL6 was strongly expressed (3 +) in fibers undergoing to atrophy (about 50% of fibers), and a weak signal (±) was present in healthy fibers. Conversely in OA patients, IL6 was uniformly expressed (2 +/3 +) in all tissues examined (Fig. 1a). Since IL6 expression is epigenetically regulated and no data are available on muscle acetylation pattern in OA an OP condition, we observed H4 acetylation state in the same tissues [28, 29].

### Histone H4 is acetylated in OP muscle tissues

**Fig. 2:**
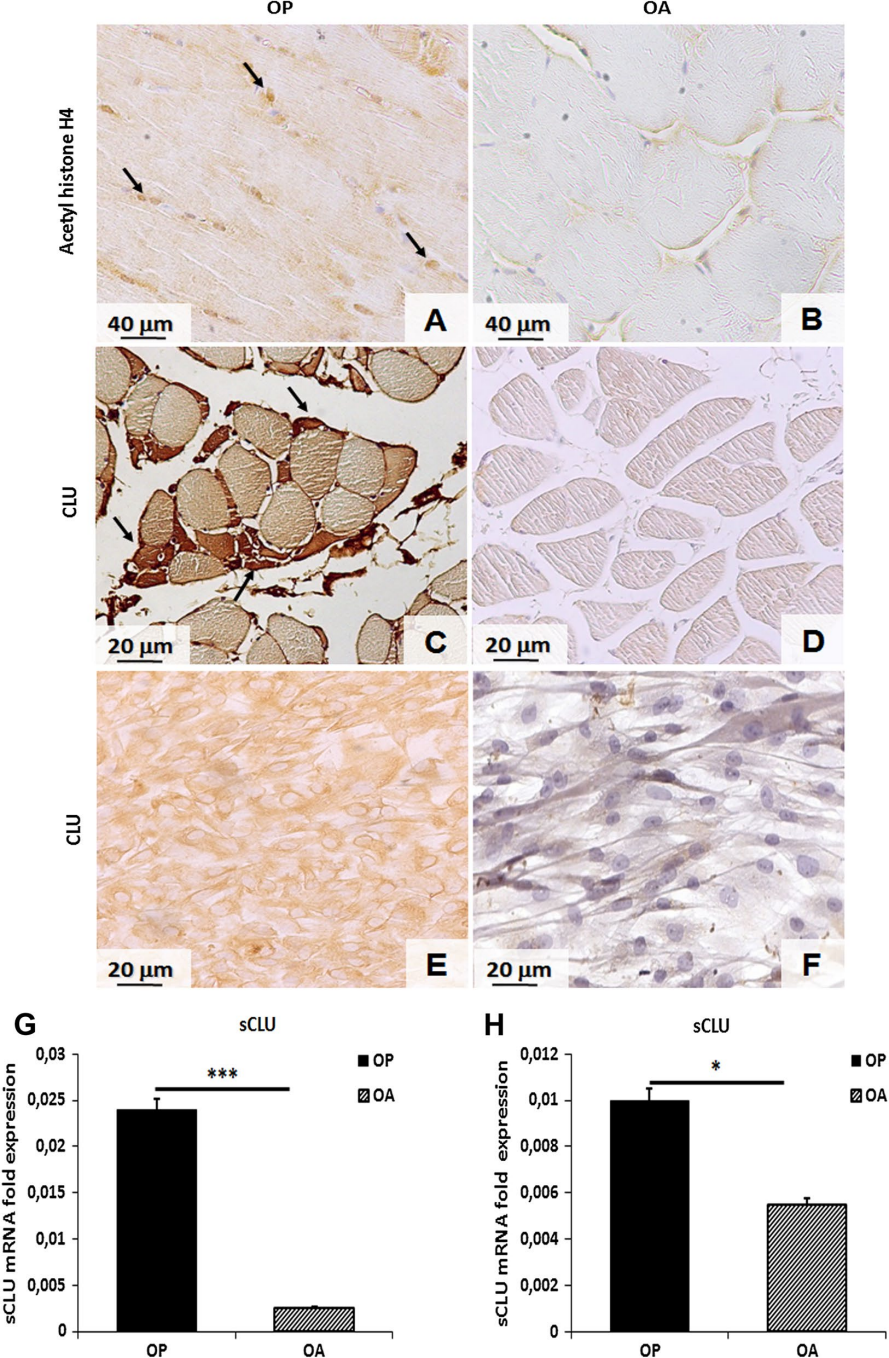
Acetyl histone H4 and CLU expression in OP and OA muscle tissue and myoblast isolated from the same patients: acetyl histone positivity determined by IHC in OP (**a**) and OA (**b**) muscle tissues. A strong immunostaining was observed in 45% of nuclei of OP tissues (p < 0.001) indicating the active DNA transcription in the attempt to repair muscle damage. On the contrary only 1% of the nuclei of OA tissues were positive. Immunostaining of CLU was performed both in muscle tissue and in isolated myoblasts of OP (**c**–**e**) and OA (**d**–**f**) patients (arrows indicate CLU positivity in degenerated fibers of OP muscle tissue). qRT-PCR analysis of sCLU expression was performed in OP-OA muscle tissues (**g**) and in OP-OA isolated myoblasts (**h**). The IHC, ICC and qRT-PCR analysis showed a strong CLU expression in OP muscle tissue and isolated myoblasts (*** p < 0.001; * p < 0.5)

Immunohistochemical reactions were evaluated by assigning a score from 0 to 3 based on positive cells number (out of a total of 500 cells in randomly selected regions). The genome-wide status of histone acetylation (associated with an active chromatin state) has not been studied so far in quiescent or activated satellite cells. Therefore, we characterized the level of histone H4 acetylation as indicator of gene transcription activity in muscle tissues from OP and OA patients [30, 31]. In OP muscle section we found a strong H4 acetylated signal in 45% of the nuclei observed, indicating a potential gene specific transcription activity (Fig. 2a). Conversely in OA patients, only a few nuclei (1%) were found positive for acetylated histone H4 in all the tissues observed (20 out of 20, p < 0.001) (Fig. 2b).

### CLU is strongly expressed in degenerated fibers of OP patients

The presence of CLU was evaluated by IHC in OP and OA muscle tissues. CLU expression was strong in the degenerate fibers of *vastus lateralis* biopsy samples of OP patients; in fact, CLU was strongly expressed (3 +) in fibers with a diminished diameter as compared to healthy muscle fibers from OP and OA (Fig. 2c). In the latter CLU expression was found diffusely and weakly expressed (1 +) as compared to OP (p < 0.001) (Fig. 2d). It is known that CLU could be also released as a circulating extracellular chaperone. Therefore, in order to verify whether its presence in muscle tissues was due to an endogenous production of the protein or to an intracellular uptake, mRNA was extracted from muscle tissues and from isolated myoblasts from the same patients as previously described. sCLU expression was determined by qRT-PCR and resulted more expressed in OP than OA patients (p < 0.001). This result confirms a potential involvement of the secreted-cytoplasmic isoform of CLU in these two different pathologies (Fig. 2g). In order to study the effect of CLU on proliferation and differentiation in OP and OA, myoblasts were isolated from muscle tissues of the same patients.

### CLU is overexpressed in OP myoblasts

Myoblasts obtained were expanded and ICC was performed in order to assess CLU expression at cellular level in the two groups. We observed that CLU is more expressed in OP (Fig. 2e) as compared to OA myoblasts (Fig. 2f). CLU was localized only in the cytoplasm of OP myoblast confirming the results obtained on different patients tissues (Fig. 2c, d). The different expression of sCLU in the two pathologies is shown in Fig. 2h; results obtained are in agreement with data on protein expression previously observed by IHC (Fig. 2a, b), confirming that OP patients express higher level of CLU as compared to OA (p < 0.05).

### CLU affects the proliferative rate of OP and OA myoblasts

**Fig. 3:**
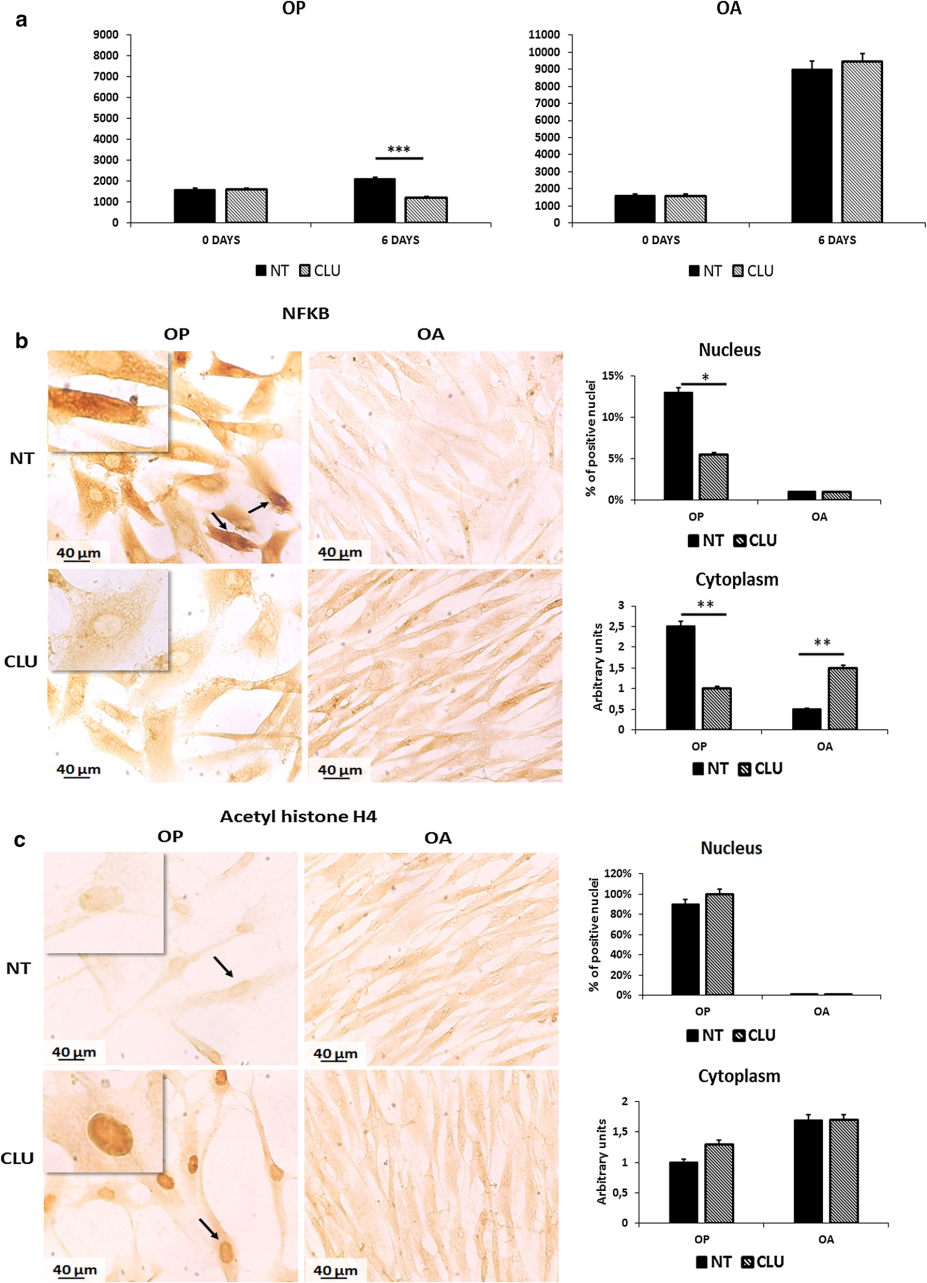
Effect of recombinant CLU on OP and OA myoblasts: **a** effect of CLU conditioning on OP and OA myoblasts proliferation. Cells were seeded and 24 h after seeding recombinant CLU was added. Only OP myoblasts showed a decrease of proliferation after 6 days of treatment (CLU vs NT: p < 0.001) (NT: untreated; CLU: treated with recombinant CLU). **b** ICC analysis of NFKB expression in untreated and CLU treated OP and OA myoblasts. CLU conditioning determined a decrease of positive nuclei for NFKB expression, only in OP myoblasts (CLU vs NT: p < 0.001). Nuclei are indicated by arrows; respectively, positive nuclei for staining in NT cells and negative nuclei in CLU treated cells. **c** ICC analysis of acetyl histone H4 expression in untreated and CLU treated OP and OA myoblasts. In this case, CLU conditioning determined a significant increase of histone H4 acetylation levels in OP treated myoblasts (CLU vs NT: p < 0.05). No significant modulation occurred in OA cells. Nuclei are indicated by arrows; respectively, weakly positive nuclei for staining in NT cells and highly positive nuclei in CLU treated cells. (***p < 0.001; *p < 0.05; **p < 0.01)

To better understand the role of CLU in the two diseases, we performed in vitro studies to evaluate the effect on myoblasts proliferative rate, isolated from OP and OA muscle tissues. Cells were seeded and 24 h after seeding recombinant CLU (2 μg/ml) was added to OP and OA myoblasts as described in Materials and methods; cultures without CLU were used as controls. First, we noticed that untreated OA myoblasts proliferated more than untreated OP cells. Furthermore, CLU affected the proliferative capacity of OP myoblasts (p < 0.001 after 6 days of treatment). CLU treated OP myoblasts showed a decrease of proliferation, unlike OA myoblasts, which did not show an effect on proliferation due to CLU treatment (Fig. 3a).

In order to define the role of CLU in the activation of nuclear factors involved in proliferation, we evaluated the potential activation of NFKB in myoblast conditioned for 6 days with CLU, in order to clarify the long lasting CLU conditioning effect on OP and OA myoblasts (Fig. 3b). In OP untreated myoblast we observed 13% of positive nuclei for NFKB staining and a strong positivity (2 +) in the cytoplasm, suggesting a high amount of the inactive protein. Conversely in OP myoblasts treated with CLU we noticed a decrease of the number of positive nuclei (5.5%) suggesting the lack of activated NFKB protein after treatment (p < 0.05) and concomitantly we observed also a decrease of the cytoplasmic signal (p < 0.01). Differently, in OA untreated myoblasts we observed a diffuse and weak localization of NFKB in the cytoplasm. After 6 days of conditioning an evident increase of NFKB expression in the cytoplasm was observed (p < 0.01).

### CLU influences histone acetylation pattern

Epigenetic changes in histone acetylation modulate gene transcription and determine cell fate. Hence, we investigate in OP and OA myoblasts the potential effect of this pleiotropic protein on the acetylation pattern by ICC, with a specific antibody recognizing acetylated histone H4. As shown in Fig. 3, OP untreated myoblasts displayed 90% of positive nuclei for acetyl histone H4 staining (2 +), confirming the results observed in OP tissues (Fig. 2a). In CLU conditioned cultures for 6 days we observed an evident increase of histone H4 acetylation level after 6 days of treatment in 100% of treated cells, suggesting an involvement of CLU in the epigenetic regulation of DNA transcription. Conversely untreated and treated OA myoblasts did not display positivity in the nucleus. The increase of histone H4 acetylation in myoblast and satellite cells has been pointed out as an indicator of differentiation and genes transcription, especially of MyoD related genes [32]. Therefore, in cultured myoblats from OP and OA patients we evaluated the effect of CLU conditioning on a myoblast terminal differentiation marker, MYOG.

### CLU modulates MYOG expression

**Fig. 4:**
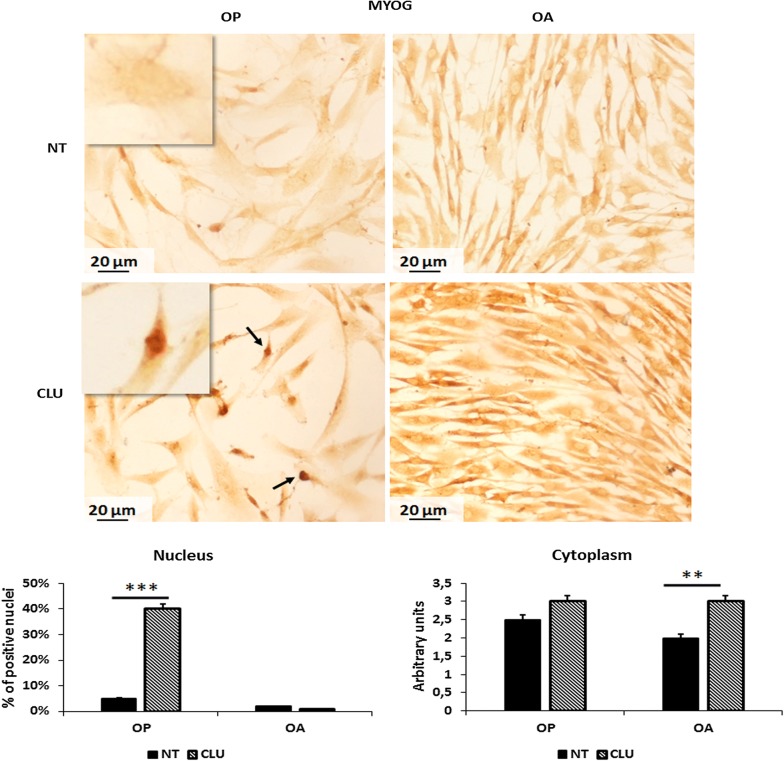
MYOG expression in untreated and CLU treated OP and OA myoblasts: An increase of positive nuclei for MYOG expression was observed in OP myoblasts treated for 6 days with CLU (CLU vs NT: p < 0.001). In OA myoblasts, we noticed an increase of MYOG expression only in the cytoplasm, as inactive form, after 6 days of treatment (CLU vs NT: p < 0.01). Nuclei are indicated by arrows; respectively, negative nuclei for staining in NT cells and positive nuclei in CLU treated cells. (***p < 0.001; **p < 0.01)

MYOG belongs to a group of myogenic regulatory proteins whose expression determines commitment and differentiation of satellite cells into skeletal muscle. Therefore, it is strongly involved in terminal differentiation of myoblasts and muscle waste. Cultures were incubated for 6 days in presence or absence of CLU and MYOG expression was evaluated by ICC. As shown in Fig. 4, in OP untreated myoblasts MYOG was expressed in the cytoplasm (1 +/2 +) and only 5% of cells displayed MYOG nuclear positivity. Following the treatment with CLU, MYOG expression was increased in the cytoplasm (2 +/3 +) and the percentage of cells MYOG positive in the nucleus were strongly increased up to 40% (p < 0.001). Moreover we observed an higher percentage of apoptotic cells as compared to untreated cells, as previously observed in proliferation assays by dye exclusion test. It is interesting to note that after 6 days of exposure to CLU, myoblasts were morphologically more differentiated as compared to untreated cells of the same experimental group. Conversely in untreated OA myoblast, MYOG was expressed and localized in the cytoplasm (1 +/2 +) and only 2% of cells presented nuclear positivity. After CLU treatment, MYOG expression was increased (2 +) in the cytoplasm but 100% of nuclei were negative for MYOG specific staining. These data indicate that the different action of CLU on MYOG activation depends on specific intracellular *milieu*.

### CLU silencing restores the proliferative capability of OP myoblasts

**Fig. 5:**
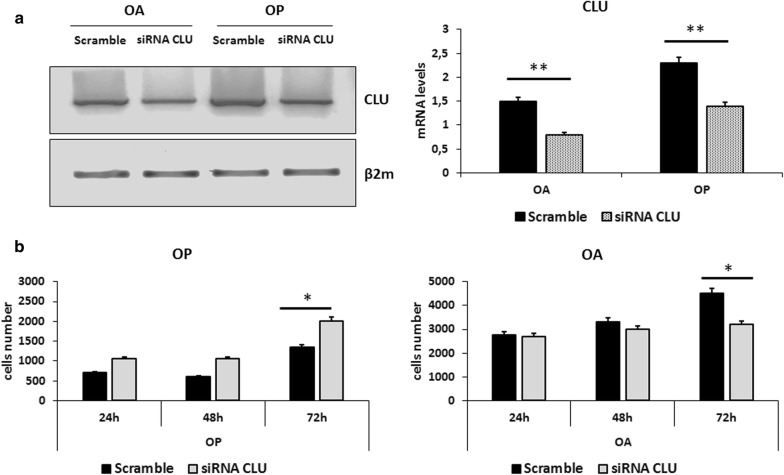
Effect of CLU silencing on OP and OA myoblasts: **a** RT-PCR analysis of CLU expression in OA and OP myoblasts transfected with CLU siRNA. The efficiency of CLU silencing was confirmed by RT-PCR. The transfection determined a strong down-regulation of CLU expression both in OA and in OP cells (p < 0.01). **b** Effect of CLU silencing on OP and OA myoblasts proliferation. Cells were seeded and 24 h after seeding cells were transfected with CLU siRNA. CLU siRNA transfection determined an increase of proliferation only in OP myoblasts (siRNA vs scramble: p < 0.05); conversely, in OA myoblasts we noticed a decrease of proliferation 72 h after transfection (siRNA vs scramble: p < 0.05). (**p < 0.01; *p < 0.05)

After studying the effect of exogenous CLU treatment on OP and OA myoblasts, we wanted to evaluate CLU gene silencing effect on the proliferation rate of these cells. Cells were seeded and after an overnight culture they were transfected with specific sequences for CLU gene and non-specific sequences as transfection control (see “Methods” section). The greatest effect was observed 72 h after transfection both in OP and OA: as shown in Fig. 5b, CLU siRNA transfection determined in OP myoblasts an increase of proliferation, as compared to scramble (p < 0.05). Conversely in OA myoblasts we noticed a decrease of proliferation (siRNA vs scramble: p < 0.05).

### CLU is involved in tissue damage repair in OP disease

**Fig. 6:**
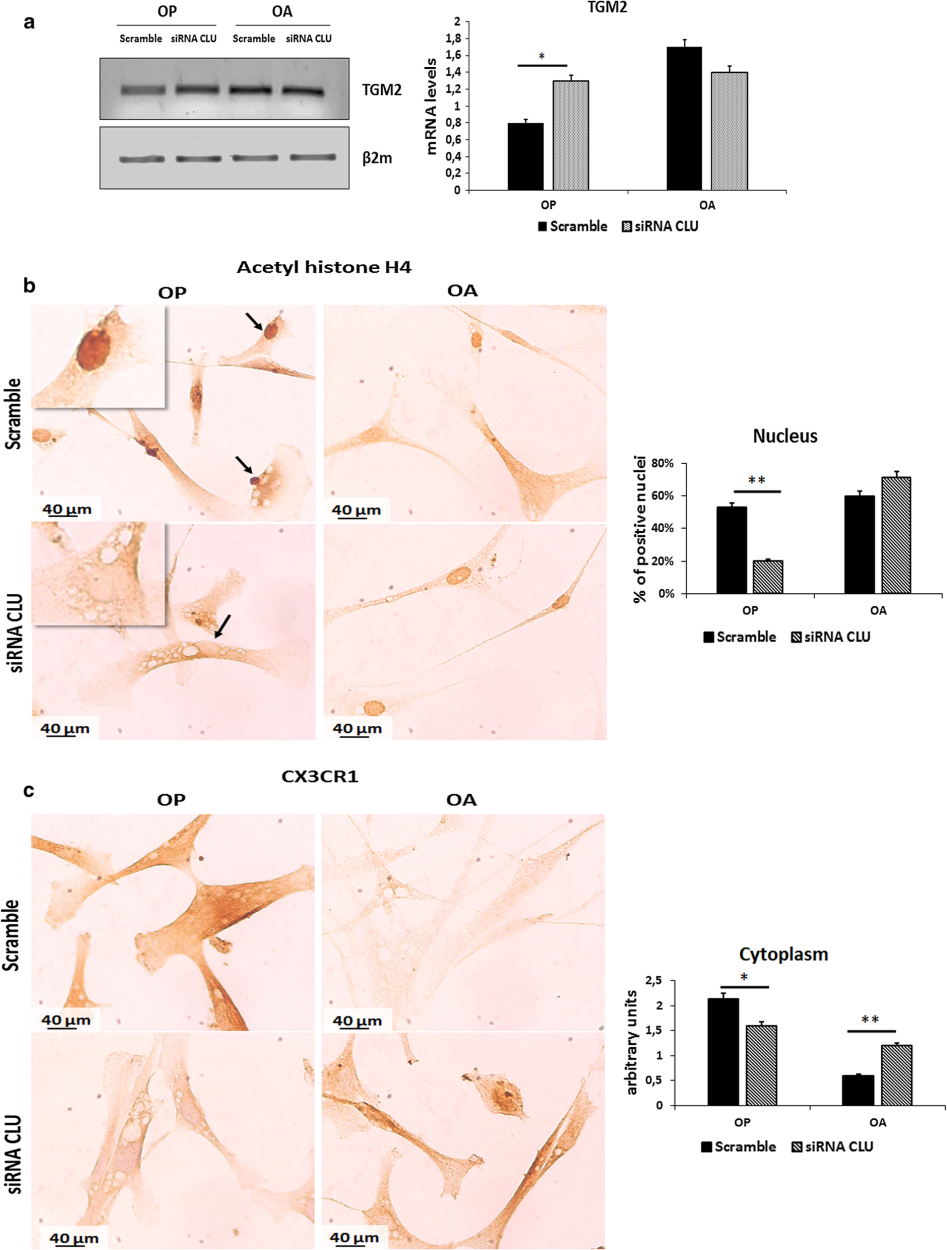
RT-PCR analysis of TGM2 expression and ICC analysis of acetyl histone H4 and CX3CR1 in OP and OA myoblasts transfected with CLU siRNA: **a** RT-PCR analysis of TGM2 expression. CLU silencing determined an increase of TGM2 expression only in OP myoblasts (siRNA vs scramble: p < 0.05); no modulation occurred in OA myoblasts. **b** ICC analysis of acetyl histone H4 in OP and OA myoblasts transfected with scramble and CLU siRNA. CLU silencing determined a strong down-regulation of positive nuclei for acetyl histone H4 expression only in OP myoblasts (siRNA vs scramble: p < 0.01); no significant modulation occurred in OA myoblasts. Nuclei are indicated by arrows; respectively, positive nuclei for staining in Scramble cells and negative nuclei in CLU siRNA treated cells. **c** ICC analysis of CX3CR1 in OP and OA myoblasts transfected with scramble and CLU siRNA. In this case, CLU silencing determined CX3CR1 cytoplasmic expression down-regulation in OP myoblasts (siRNA vs scramble: p < 0.05) and an increase of CX3CR1 cytoplasmic expression in OA myoblasts (siRNA vs scramble: p < 0.01). (*p < 0.05; **p < 0.01)

It is known that OP patients are characterized by degenerated and atrophic muscle fibers. In order to evaluate a potential role of CLU in tissue damage repair, we analyzed the expression of Transglutaminase 2 (TGM2), that plays an important role in anti-apoptotic signalling pathways, and is involved in many cellular processes, including various physiological responses and pathological states, thus contributing to the protection of many forms of tissue damage, wound healing and inflammation [33, 34]. Cells were seeded and transfected with CLU siRNA; cells transfected with scramble were used as control. 48 h after transfection, cells were collected for RNA extraction and for TGM2 gene expression analysis through RT-PCR (see “Methods” section). As shown in Fig. 6a, CLU silencing through siRNA transfection determined an increase of TGM2 expression only in OP myoblasts (p < 0.05). No significant modulation occurred in OA myoblasts. Based on these results, CLU silencing could restore the ability to repair tissue damage only of OP muscle cells.

### CLU silencing affects DNA acetylation and modulates a mediator of the inflammatory response, CX3CR1

Our previous results obtained from exogenous CLU conditioning on OP and OA myoblasts showed a potential involvement of CLU in the epigenetic regulation of DNA, determining a strong increase of histone H4 acetylation in OP myoblasts after 6 days of treatment. We studied OP and OA acetylation pattern also after siRNA transfection for CLU silencing (see “Methods” section). As shown in Fig. 6b, CLU silencing seemed to invert CLU conditioning effect only in OP myoblasts, determining a decrease of positive nuclei for acetyl histone H4 staining, from 53 to 20.2% (p < 0.01), as compared to scramble. No significant modulation occurred in OA transfected cells (Fig. 6b).

OP and OA are two diseases characterized by a strong inflammatory component, especially OA, whose muscle fibers present a strong expression of IL6 (see Fig. 1a), main inflammatory cytokine.

In order to study a possible involvement of CLU in the modulation of the inflammatory response, we analyzed, though ICC, the effect of CLU on CX3CR1 expression. CX3CR1 is a receptor highly expressed on Th1 activated T cells and other activated cell types that, through interaction with its ligand, induces chemotaxis of circulating monocytes and selective recruitment of Th1 lymphocytes responsible of active cronic inflammation [35, 36]. As shown in Fig. 6c, OP myoblasts silenced for CLU gene, showed a decrease of CX3CR1 receptor cytoplasmic expression (p < 0.05). Conversely, CLU silencing determined a strong increase of CX3CR1 expression compared to the scramble (p < 0.01) in OA myoblasts. These results seemed to confirm the potential involvement of CLU in the epigenetic regulation of DNA through histone acetylation and in the inflammatory response.

## Discussion

OP and OA are extremely frequent among elderly people, and their impact on life quality makes them of high social health relevance [37, 38]. The identification of new markers and metabolic targets involved in OP and OA processes is an objective of extreme interest for the discovery of new biological drugs and for the improvement of therapeutic strategies [39]. For the first time, we provided evidences that CLU is strongly expressed in degenerated fibers of OP patients as compared to OA. Moreover, we observed that CLU in OP could be involved in the modulation of histone acetylation and myoblasts terminal differentiation. The latter effect includes inhibition of NFKB nuclear localization and MYOG activation by its nuclear translocation. In fact, the higher level of histone acetylation could be related to the increased expression of genes involved in the activation of satellite cells, to their proliferation and migration towards the atrophic fibers, where they merge and give rise to new regenerated fibers.

Firstly, we characterized the distribution and the expression of the proinflammatory cytokine IL6 in the muscle tissues from the two experimental groups. We observed differences in IL6 expression depending on the severity of the disease and the type of pathology. We found that IL6 was uniformly expressed in OA muscle fibers confirming the strong and diffuse inflammatory state of this pathology. On the contrary, in muscle tissues of OP patients we found a higher expression of IL6 in atrophic fibers and a weaker expression in healthy fibers. According to VanderVeen et al., high circulating levels of IL6 and other proinflammatory citokines, disrupt mitochondrial homeostasis, leading to mitochondria dysfunction and to muscle mass loss during cancer cachexia; in fact, mitochondrial disfunction in skeletal muscle negatively regulates muscle mass. Our previous studies evidenced a link between IL6 and CLU overexpression in highly aggressive tumors, suggesting a possible connection of IL6 derived inflammation with CLU overexpression and neoplastic transformation [14]. We observed that CLU expression was closely related to IL6 presence in muscle tissues. In fact, in OA patients we observed a strong diffused IL6 expression in the sarcoplasm of skeletal muscle tissue, concurring with the chronic inflammatory status of this pathology. In the same patient group we found also a moderate and diffuse presence of CLU, in agreement with data previously published [40]. An overlapping strong expression of IL6 and CLU in fully degenerated or degenerating fibers in muscle tissues of OP patients was noted. Moreover, our in vitro studies on cultures conditioned with CLU have demonstrated a strong involvement of CLU in the downmodulation of cell proliferation and cell death induction after 6 days of treatment, suggesting a potential role of CLU in affecting myoblasts viability. The increased cell proliferation was accompanied also by NFKB downregulation and relocalization in the cytoplasm of OP myoblasts, suggesting a potential network among CLU-NFKB and proliferative arrest. We found a differential histone H4 acetylation pattern in OP and OA tissues. The increased histone H4 acetylation, induced by CLU treatment in OP myoblasts, associated with an active chromatin state, was correlated with a proliferative arrest, as demonstrated by an evident decrease of proliferation and the induction of differentiation exerted by CLU, in OP myoblasts only. The increased histone acetylation during differentiation correlates with the transcription activity of MyoD target genes [41], and thus suggest that histone acetylation is increased in a subset of genes important for myogenic differentiation.

Accordingly, we observed that CLU affected MYOG expression and localization. In fact the observations on MYOG distribution in untreated OA myoblast indicated that MYOG is located in the cytoplasm only, demonstrating that in OA cells this protein is probably present in an inactive state. CLU is able to induce a light increase of MYOG expression in OA myoblasts only in the cytoplasm. In untreated OP myoblasts, MYOG is present in the cytoplasm as a reservoir and the 80% of nuclei were negative. In OP myoblast cultured in CLU conditioning medium for 6 days, we observed that CLU was able to strongly trigger MYOG translocation from cytoplasm to the nucleus affecting its expression and activation, inducing, as observed phenotipically, terminal differentiation and senescence accompained by cell death. NFKB decrease and inactivation is strongly associated with a decreased proliferation rate in OP, confirming that CLU is operating towards a terminal differentiation and a premature aging state. These results are in agreement with the presence of a strong CLU expression in degenerated fibers as observed in vivo on the tissues of the same patients. Results obtained from CLU silencing experiments confirmed the role of CLU observed also when conditioning OP and OA isolated myoblasts with exogenous CLU. First of all, we noticed that CLU silencing seemed to restore proliferating ability in OP myoblasts only. Conversely, in OA myoblasts, 72 h after transfection, CLU silencing determined a decrease of proliferation. Silencing experiments showed also how the DNA acetylation pattern is reversed in OP myoblasts only. Indeed, OP transfected cells showed a decrease of histone H4 acetylation as compared to OA transfected myoblasts. These data highlights the negative role played by CLU in the osteoporotic disease. Our data evidenced a strong CLU influence on inflammation, through the modulation of CX3CR1, a receptor that, when activated by its ligand causes the chemotaxis of monocytes and the recruitment of Th1 lymphocytes, thus influencing a chronic and active inflammatory state. Hence, CLU silencing in OP strongly reduced the expression of CX3CR1, indicating the potential of CLU to amplify the immune response in this pathology. These data point out a strong correlation between CLU and chronic inflammation typical of the OP-OA condition. In addition, data on CLU silencing in OP demonstrated the effect of CLU also in the activation and expression on TGM2, negatively influencing the tissues damage response. According to the results obtained, CLU, which is strongly expressed in OP patients, seems to exercise a negative effect on the onset and progression of the osteoporotic disease, since its downregulation protects from inflammatory events and restores tissue damage repair ability. Taken together these data strongly suggest that CLU could influence the satellite cells differentiation, inducing a proliferative pulse, cell reset and MYOG activation in OP myoblasts only. Since in muscle tissues of OP patients, as previously published [42], there is a low reservoir of resident satellite cells, the high level of CLU could induce satellite cells to differentiate, exhausting the satellite cells pool available to repopulate the muscle fibers damaged and leading to a severe sarcopenia. Data obtained in OP myoblasts suggest a possible involvement of CLU in osteoporotic disease, in participating to the loss of satellite cells pool and in massive induction of terminal differentiation and premature senescence. These processes eventually lead to a premature degenerative process and aging, pointing out a potential role of CLU as a new OP diagnostic marker for muscular degeneration and a potential target for specific therapeutic intervention in OP related sarcopenia.

## Conclusions

In the present study we observed that CLU, a pleiotropic protein involved in cellular senescence, aging and various age-related diseases, including neurodegeneration, inflammation, vascular damage, diabetes and tumourigenesis, is strongly expressed in the degenerated muscular fibers undergoing atrophy in OP patients. The long lasting somministration of CLU on human isolated myoblasts in vitro induces a proliferative arrest, a modulation of histone acetylation and the nuclear activation of the terminal differentiation marker, MYOG. In addition CLU silencing is able to reduce the expression of CX3CR1, downregulating the local inflammatory response. These data suggest a potential involvement of CLU in the modulation of the inflammation state and in the induction of the premature senescence of osteoporotic myoblasts. Depleting satellite cells pool, causes the state of sarcopenia associated to the osteoporotic disease. Although further studies are warranted to elucidate the exact role of CLU, the present study highlighted the potential action of CLU in osteoporotic disease, suggesting new clinical approaches and strategy for intervention.

## Abbreviations

OP: osteoporosis
OA: osteoarthritis
CLU: Clusterin
sCLU: secreted Clusterin
nCLU: nuclear Clusterin
IL6: interleukin 6
IHC: immunohistochemistry
ICC: immunocytochemistry
BMD: bone mineral density
BMI: body mass index
HHS: Harris hip score
JSN: joint space narrowing
NFKB: nuclear factor kappa-light-chain-enhancer of activated B cells
TGM2: transglutaminase 2
CX3CR1: CX3C chemokine receptor 1
